# The Medical Register

**Published:** 1909-02-13

**Authors:** H. E. Allen

**Affiliations:** Registrar. General Council of Medical Education and Registration of the United Kingdom, 299 Oxford Street, London, W.


### THE MEDICAL REGISTER.

Dear Sir,—A very searching inquiry is now being made as to the accuracy of all names and addresses on the Medical Register under the following Section (XIV.) of the Medical Act, 1858:

*XIV. It shall be the duty of the Registrars to keep their respective Registers correct in accordance with the provisions of this Act, and the Orders and Regulations of the General Council, and to erase the names of all registered persons who shall have died, and shall from time to time make the necessary alterations in the addresses or qualifications of the persons registered under this Act; and to enable the respective Registrars duly to fulfil the duties imposed upon them it shall be lawful for the Registrar to write a letter to any registered person, addressed to him according to his address on the Register, to inquire whether he has ceased to practise, or has changed his residence, and if no answer shall be returned to such letter within the period of six months from the sending of the letter it shall be lawful to erase the name of such person from the register; provided always that the same may be restored by direction of the General Council should they think fit to make an order to that effect.*

On February 1 a circular of inquiry was posted to every registered practitioner, *excepting only* officers of the Navy, Army, and Indian Medical Services whose names appear in the Navy and Army Lists.

It is of vital importance to all registered practitioners who have not received an inquiry in course of post that they should immediately communicate with this office, as in the event of no communication being received from them, it will *be lawful to erase their names from the Register*, according to the Act, and then very serious disabilities will be incurred.

Yours faithfully,

H. E. Allen, Registrar.

General Council of Medical Education and Registration of the United Kingdom, 299 Oxford Street, London, W.
February 3, 1909.

